# R‐Ras deficiency does not affect papain‐induced IgE production in mice

**DOI:** 10.1002/iid3.168

**Published:** 2017-05-11

**Authors:** Laura Kummola, Zsuzsanna Ortutay, Maria Vähätupa, Stuart Prince, Hannele Uusitalo‐Järvinen, Tero A.H. Järvinen, Ilkka S. Junttila

**Affiliations:** ^1^ Faculty of Medicine and Life Sciences University of Tampere Tampere Finland; ^2^ Departments of Ophthalmology and Orthopaedics & Traumatology Tampere University Hospital Tampere Finland; ^3^ Fimlab Laboratories Tampere Finland

**Keywords:** Allergology, dendritic cells, IgE, knock‐out mice, R‐Ras

## Abstract

**Introduction:**

R‐Ras GTPase has recently been implicated in the regulation of immune functions, particularly in dendritic cell (DC) maturation, immune synapse formation, and subsequent T cell responses.

**Methods:**

Here, we investigated the role of R‐Ras in allergen‐induced immune response (type 2 immune response) in Rras deficient (R‐Ras KO) and wild type (WT) mice.

**Results:**

Initially, we found that the number of conventional DC's in the lymph nodes (LNs) was reduced in R‐Ras KO mice. The expression of co‐stimulatory CD80 and CD86 molecules on these cells was also reduced on DC's from the R‐Ras KO mice. However, there was no difference in papain‐induced immune response between the R‐Ras WT and KO as measured by serum IgE levels after the immunization. Interestingly, neither the DC number nor co‐stimulatory molecule expression was different between WT and R‐Ras KO animals after the immunization.

**Conclusions:**

Taken together, despite having reduced number of conventional DC's in the R‐Ras KO mice and low expression of CD80 on DC's, the R‐Ras KO mice are capable of mounting papain‐induced IgE responses comparable to that of the WT mice. To our knowledge, this is the first report addressing potential differences in in vivo allergen responses regulated by the R‐Ras GTPase.

## Introduction

R‐RAS is a small GTPase, a member of the extensive Ras superfamily, and shows about 55–60% amino acid identity with the classical RAS proteins (H‐RAS, K‐RAS, N‐RAS) 1, 2. Despite the close structural similarity to other members of the Ras family, the function of R‐Ras is distinct from other Ras proteins. While all other members of the Ras family may cause malignant transformation, R‐Ras has very little or no transforming activity. R‐Ras plays a role in several cellular processes such as integrin signaling 3, 4, actin cytoskeleton organization 5, membrane ruffles, cell spreading 6, 7, cell adhesion 4, 8, and migration 9. Concerning leukocytes, *Rras^−/−^* mice show impaired T cell trafficking 10 while murine macrophages expressing constitutively activated R‐Ras show elevated phagocytotic activity 11.

R‐RAS can regulate inflammation 12, 13, 14, 15. Dendritic cells (DC's) from R‐Ras deficient animals have reduced ability to prime T cell responses due to unstable immune synapse formation 14. In experimental autoimmune encephalomyelitis model (EAE), *Rras^−/−^* mice showed attenuated disease course likely due to elevated numbers of peripheral tolerogenic and regulatory T cells (Tregs) 15. Recently we showed that R‐Ras is required for tumor development and progression in inflammation‐dependent skin cancer model 13. Although R‐Ras was present only in the blood vessels and in skin, it was required both for the induction of pro‐inflammatory cytokine production and for the extravasation of inflammatory cells to the skin 13.

Given the key role of DC's in adaptive immune responses and the role of R‐Ras in regulating DC functions 14, 15 we wanted to know whether type2 immune response is regulated by R‐Ras in vivo. We found that despite the fact that untreated R‐Ras KO had decreased number of conventional DC's and the DC's show reduced expression of CD80/CD86 costimulatory molecules, these mice were capable of mounting a normal allergen‐induced response after papain immunization.

## Methods

### Mice, immunizations, ELISA, cell culture, flow cytometry

R‐Ras KO (= Rras^Gt(OST24882)Lex^) mice (C57BL/6) have been described 16. Animal experiment protocol was approved by Finnish National Animal Experiment Board (Permit: ESAVI/4738/04.10.07/2014). Administration of papain was performed as a modified version of the protocol described earlier by Sokol et al. 17. Mice were injected subcutaneously either with PBS alone or PBS containing 0.5 mg papain (Merck Millipore, Darmstadt, Germany) on days 0 and 14. Blood samples were collected from the tail vein on day 0. Mice were euthanized on day 16 or day 21, and spleens and LNs were harvested. Blood was collected by cardiac puncture into serum blood tubes (BD, Franklin Lakes, NJ). IgE ELISA was from eBioscience (Santa Clara, CA).

Spleens and pooled LNs were incubated in 75 μg/ml Liberase DL and 10 U/ml DNase (Roche, Basel, Switzerland) for 30 min at 37°C. DC's were enriched with MACS Pan Dendritic Cell Isolation Kit (Miltenyi Biotec, Bergisch Gladbach, Germany). Alternatively, spleen cells were gradient centrifuged with Histopaque®‐1077 (Sigma–Aldrich, St. Louis, MO).

Antibodies were (clones in parenthesis): CD3‐APC (145‐2C11) or ‐APC‐eFluor780 (17A2), CD4‐FITC or ‐APC‐eFluor780 (both GK1.5), CD8‐PerCP‐Cy5.5 or ‐APC‐H7 (both 53–6.7), B220‐Pe‐Cy7 or ‐APC‐eFluor780 (both RA3‐6B2), CD11c‐PE‐Cy7 (N419), F4/80‐APC‐eFluor780 (BM8), MHCII‐FITC (M5/114.15.2), CD80‐PerCP‐eFluor(16‐10A1), CD86‐APC (GL1), and OX40L‐PE (RM134L) (all from eBioscience). Analysis was done by FACS Canto II (BD, Franklin Lakes, NJ) and FlowJo (Tree Star, Ashland, OR). Geometrical means and standard error of means are indicated.

### Real‐time PCR, statistical analysis

Spleens were harvested into RNAlater (Qiagen, Hilden, Germany). mRNA was extracted by Trizol Reagent (Life Technologies, Carlsbad, CA) and RNA was converted to cDNA by Thermo Maxima First Strand cDNA Synthesis Kit (Life Technologies). FAM‐labelled Taqman probes (Applied Biosystems, Foster City, CA) for IL‐10 (Mm01288386_m1), IL‐13 (Mm00434204_m1), IL‐17a (Mm00439618_m1), IFN‐γ (Mm01168134_m1), FOXP3 (Mm00475162_m1), GATA3 (Mm00484683_m1), T‐BET (Mm00450960_m1), and RORγ (Mm01261022_m1) were used with iQ™ Supermix (Bio‐Rad Laboratories Inc., Hercules, CA) for qPCR. VIC‐labelled Eucaryotic 18S rRNA Endogenous Control (Applied Biosystems) was reference. Results were calculated by double delta method. Statistical analysis were done with Prism (GraphPad Software, Inc., La Jolla, CA) or R 18, 19 (for ELISA). *p*‐values were calculated using two‐tailed, unpaired student's *t*‐tests.

## Results and Discussion

**Figure 1:**
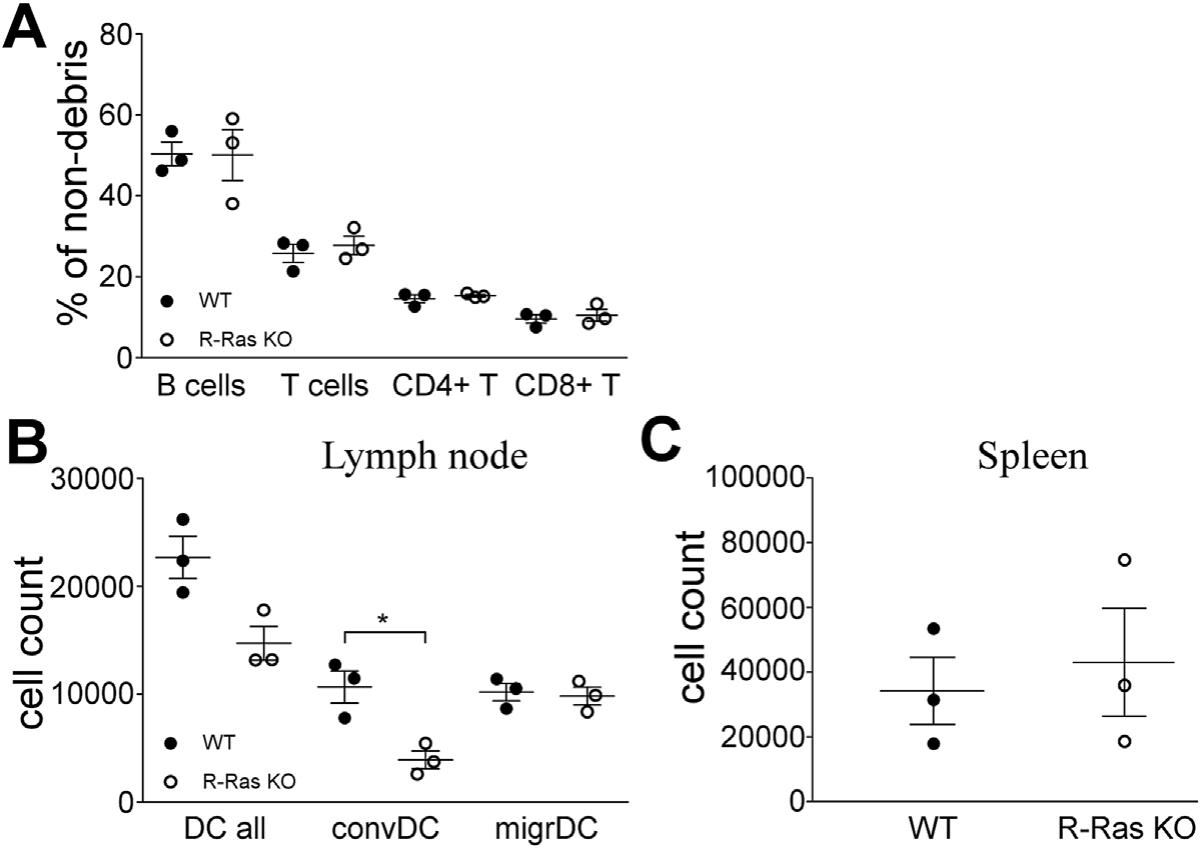
Immunophenotyping of un‐manipulated R‐Ras KO mice. (A) Lymphocyte populations of “off‐the‐shelf” wild type (WT, *n* = 3) or R‐Ras deficient (R‐Ras KO, *n* = 3) mice were compared by flow cytometry. (B and C) LNs (B) and spleens (C) were collected from untreated wild type (WT, *n* = 3) or R‐Ras deficient (R‐Ras KO, *n* = 3) mice. LNs from each mouse were pooled separately. DC's were enriched by magnetic separation (LN) or gradient centrifugation (spleens) and analyzed by flow cytometer. LN MHCII + CD11c+ (DC all) cell group includes two populations: conventional (convDC) and migratory dendritic cells (migrDC). (B) The amount of all MHCII + CD11c + dendritic cells (DC all) and their distribution between conventional (convDC) and migratory (migrDC) dendritic cells. (C) The amount of MHCII + CD11c + dendritic cells in the spleen. For statistical analysis unpaired two‐tailed *t*‐tests were used. *p < 0.05.

R‐Ras KO mice lacking functional R‐Ras protein are phenotypically similar to WT counterparts 13, 16, 20. However, DC maturation and immunological synapse formation with T cells is impaired in R‐Ras KO mice and the capability of *Rras* ^−/−^ DC's to induce allogeneic or antigen‐specific autologous T cell proliferation is reduced 14. As described 14, we found that B‐ and T cell numbers in spleen of untreated R‐Ras KO mice (*n* = 3) were normal (Fig. 1A, gating strategy in Supplementary Fig. S1A).

The less severe EAE in R‐Ras KO mice is due to a local increase in the number of Tregs and tolerogenic DC's 15. This led us to study the number and phenotype of DC's in WT and R‐Ras KO mice. Analysis of magnetically enriched conventional and migratory DC's from the LN (gating strategy in Supplementary Fig. S1C) indicated that R‐Ras KO mice had less conventional DC's than WT mice (Fig. 1B), (*p* = 0.0126), while the number of migratory DC's was similar between the two genotypes (Fig. 1B). As previously shown 14, the analysis of the enriched splenic DC's from spleen showed no difference between WT or R‐Ras KO mice (Fig. 1C, gating strategy in Supplementary Fig. S1D). We chose to exclude plasmacytoid DC's from our analysis, as they are currently considered less relevant to allergic responses: they are known producers of IFNγ upon TLR7/TLR9 activation during viral infections and autoimmune disorders 21.

**Figure 2:**
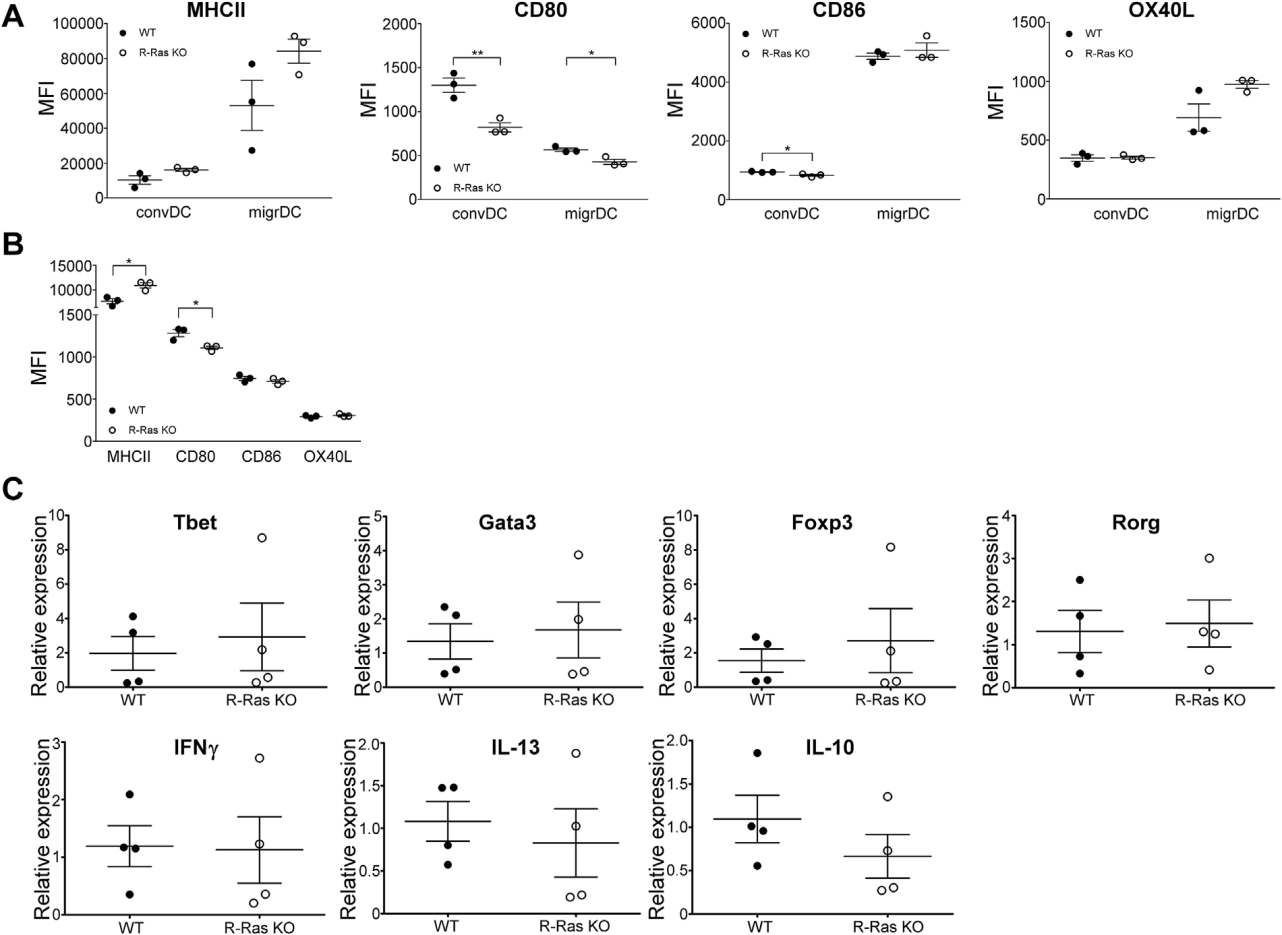
Comparison of the expression of dendritic cell surface proteins and expression of T‐helper cell transcription factors and cytokines between WT and R‐Ras KO mice. LNs (A) and spleens (B) were collected from untreated wild type (WT, *n* = 3) or R‐Ras deficient (R‐Ras KO, *n* = 3) mice and DC's were enriched as in Figure 1. (A) The expression of MHCII, CD80, OX40L, and CD86 surface proteins in conventional (convDC) and migratory dendritic cells (migrDC) from the LNs and (B) from the spleen. (C) Spleens of un‐manipulated WT and R‐Ras KO mice (*n* = 4 for each genotype) were harvested. mRNA was isolated from total splenocytes and transcribed into cDNA. The expression of Th‐specific transcription factors and cytokines were investigated with qPCR. The differences within surface protein expression (A and B) or gene expression (C) values were statistically analyzed by two‐tailed unpaired *t*‐tests. *p < 0.05, **p < 0.01. Each dot represents an individual animal. Mean values with SEM are marked in the scatter dot plots.

The strength of T cell receptor(TCR)‐engagement in cytokine‐free environment regulates T cell differentiation. Weak TCR‐signal leads to spontaneous, GATA3‐ and IL‐2‐dependent, IL‐4 production while strong TCR signal causes, ERK‐dependent, IFN‐γ production 22. The DC surface markers that take part in the DC/T‐cell interaction include MHCII (antigen presentation to TCR), CD80/CD86 (co‐stimulatory molecules associated with TCR) and OX40L (enhancement of Th2 differentiation at least in humans) 23. MHCII expression was identical in both conventional and migratory LN DC's between WT and R‐Ras KO mice (Fig. 2A). CD80 expression, in turn, was reduced in both conventional (*p* = 0.0078) and migratory (*p* = 0.0176) subsets from R‐Ras KO mice (Fig. 2A), while CD86 expression was decreased in conventional DC subset of R‐Ras KO mice (*p* = 0.0241), but not in migratory DC subset (Fig. 2A). OX40L expression was identical in conventional or migratory DC's between KO and WT mice (Fig. 2A). In spleen, MHCII expression was higher in the KO DC's (*p* = 0.0129) while CD80 expression was lower in the KO DC's (*p* = 0.0207) and, CD86 or OX40L expression were identical (Fig. 2B).

To better understand the role of R‐Ras in tuning DC activation, we analyzed the response of splenic DC's from R‐Ras deficient and WT mice to IL‐4, LPS, and poly(I:C) during overnight plate culture, which “spontaneously” activates DC's 24. Although LPS and poly(I:C) are not considered driving agents of Th2 polarization, we were interested to examine whether there was some general impairment of DC responses in *Rras* ^−/−^ mice and thus included these stimulants into the comparison.

Collectively, in splenic DC's MHCII expression is higher on DC's from R‐Ras KO mice under all tested culture conditions. The differences observed in CD80 and CD86 expression between R‐Ras KO and WT cells after overnight culture were abolished when the cells were further activated with poly(I:C), LPS or IL‐4, while OX40L was induced by poly(I:C) and LPS, but R‐Ras expression plays no role in the regulation of OX40L expression as no difference was identified between KO and WT cells (Supplementary Fig. S2A–D).

Taken together, the main difference in DC's between WT and R‐Ras KO mice is that there is significantly less conventional DC's in R‐Ras KO mice and these cells express less co‐stimulatory molecules, particularly CD80. These results suggest, that antigen presentation via MHCII is not impaired in R‐Ras DC's but the formation of immunological synapse due to decreased CD80/CD86 expression is impaired, which in turn might result in impaired CD28 signalling in T‐cells.

Since EAE in R‐Ras KO mice is less severe than in WT mice, we wanted to study if type 2 responses are affected in these mice. To analyze a possible “skew” in the steady‐state CD4 T cells in R‐Ras KO mice, we studied the mRNA expression of critical transcription factors related to helper T cell differentiation from the total splenocytes of WT and R‐Ras KO mice, but found no difference in the mRNA expression of Tbet, GATA3, Foxp3, or Rorγ (Fig. 2C, upper panel). Also, the mRNA expression of IFNγ, IL‐13, and IL‐10 was identical between WT and R‐Ras KO mice (Fig. 2C lower panel).

**Figure 3:**
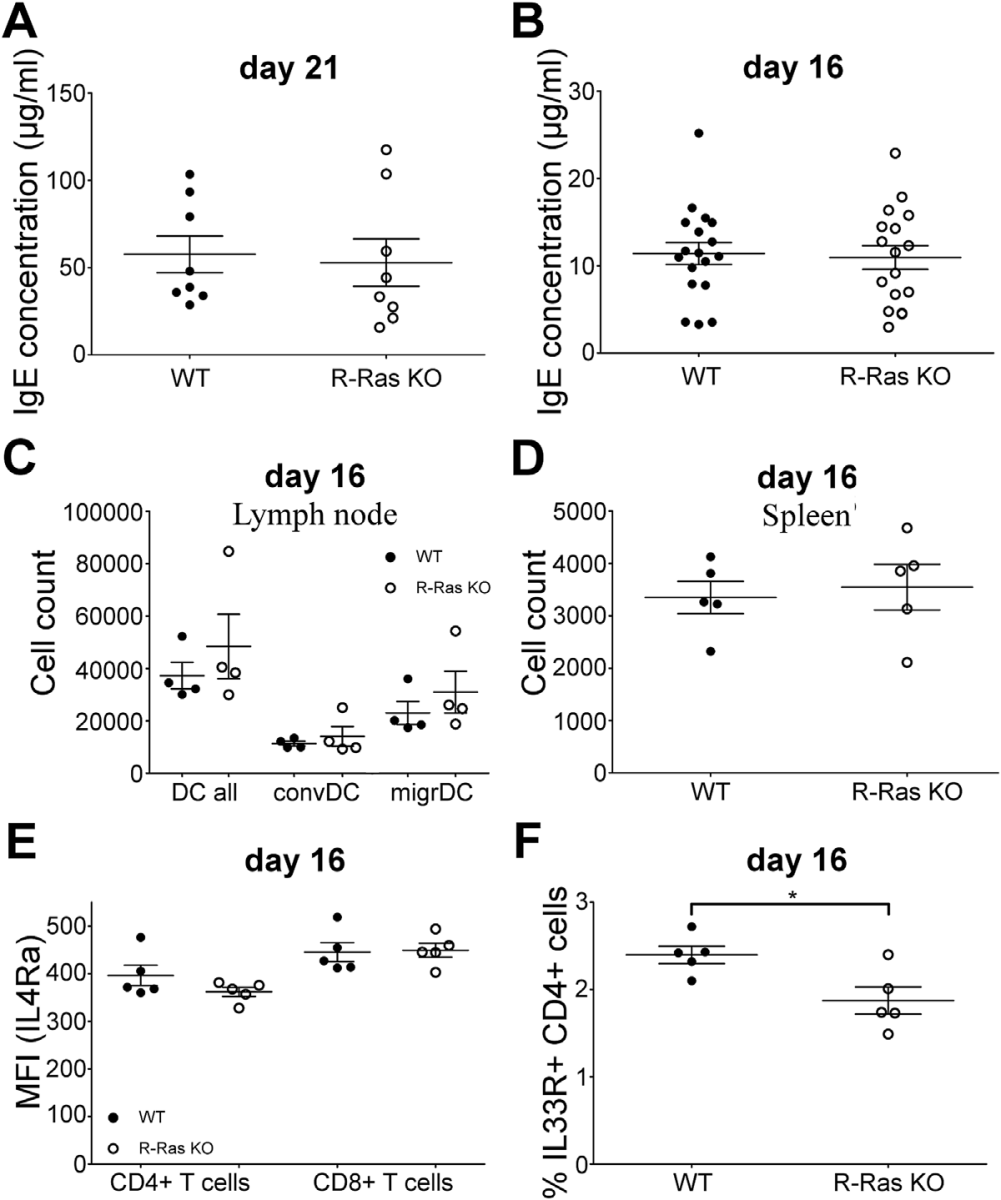
The effect of papain‐immunization on WT and R‐Ras‐deficient mice. Wild type (WT) or R‐Ras deficient (R‐Ras KO) mice were treated with papain (500 μg/animal, subcutaneous injection on day 0 and day 14). Animals were euthanized either on day 21 or day 16. IgE concentration of blood serum was measured using ELISA on (A) day 21 or (B) day 16. Experiment in A was done twice with four mice of each genotype and in B the experiment was done four times with 4–5 mice of each genotype. (C and D) LNs (*n* = 4) and spleens (*n* = 5) were collected from papain‐immunized (day 16) wild type (WT) or R‐Ras deficient (R‐Ras KO) mice and dendritic cells were enriched as in Figure 1. (C) The amount of all LN MHCII + CD11c + dendritic cells (DC all) and their distribution between conventional (convDC) and migratory (migrDc). (D) The amount of MHCII + CD11c + dendritic cells in the spleen. (E) Expression of IL‐4Rα in CD4 and CD8 T cells of WT and R‐Ras KO mice (*n* = 5 each genotype) on day 16 after papain immunization. (F) Percent of IL‐33R positive cells of CD4 T cells from WT and R‐Ras KO mice on day 16 after papain immunization. Mean values with SEM are marked in the scatter dot plots. Each dot represents an individual animal. *p < 0.05 (unpaired *t*‐tests).

To study the allergen‐induced response in R‐Ras KO mice, we utilized papain‐immunization as an in vivo model. Papain‐induced responses are mediated via migratory DC's in skin 25 and we had already observed a decrease in CD80 expression in migratory DC's (Fig. 2A) in R‐Ras KO mice. We subjected R‐Ras KO mice and WT animals (*n* = 8 for each genotype) to papain injections twice: first immunization was performed on day 0 and re‐immunization on day 14. We measured the serum level of IgE on day 0 and again when the mice were euthanized on day 21. We found no significant difference in the serum IgE levels between the two genotypes (Fig. 3A) 21 days after the first immunization. Next we wanted to rule out the possibility that the initiation phase of the type 2 response to papain might be delayed due to the impaired immune synapse formation 14 in R‐Ras KO mice. For this, we measured the IgE levels of papain‐immunized WT and KO (*n* = 17 and 18, respectively) mice only two days after the second immunization (i.e., 16 days after the first immunization). However, no difference in the serum level of IgE was found between R‐Ras KO mice and WT (Fig. 3B). The concentration of IgE was approximately 10 µg/ml on day 16, while it was ∼50 μg/ml on day 21, indicating that the allergen response was still being induced after day 16 (Fig. 3A and B).

Next, we determined the number of DC's on day 16 after the papain immunization. Unlike in unmanipulated mice, which showed a difference in the number of conventional DC's between WT and R‐Ras KO animals (Fig. 1B), we found no difference in their number (Fig. 3C) in papain‐treated animals. Similarly, the number of splenic DC's was alike in WT and R‐Ras KO mice (Fig. 3D). We also determined Th2‐associated surface markers on CD4 T cells after papain immunization. The expression of the cytokine‐binding receptor chain for IL‐4 (IL‐4Rα) is critical for CD4 T cell response to IL‐4 and is up‐regulated on CD4 cells during Th2 response. We found no difference in IL‐4Rα expression between WT or R‐Ras KO mice (*n* = 5) either in CD4 or CD8 cells (Fig. 3E). IL‐33R is another cytokine receptor chain up‐regulated in CD4 T‐cells in Th2 responses. IL‐33 can regulate “effector cytokine,” such as IL‐5 and IL‐13, expression in Th2 cells in concert with Stat5 activating cytokines, independently of antigens 26. IL‐33 has been shown to be indispensable for papain‐induced IgE production in murine lung and genital tract 27, 28. We found that the percent of IL‐33R positive cells was reduced in R‐Ras KO CD4 T cells (Fig. 3F, the gating strategy for IL‐33R is shown is supplementary Fig. S1B). This might suggest that while IL‐4 mediated events (IgE production, IL‐4Rα expression) are intact in R‐Ras KO mice, IL‐4 independent (but GATA3‐dependent) events, such as IL‐33R expression can be affected by the deletion of R‐Ras. The requirement of IL‐4 for Th2 differentiation in vivo is discussed in detail in reference 29.

**Figure 4:**
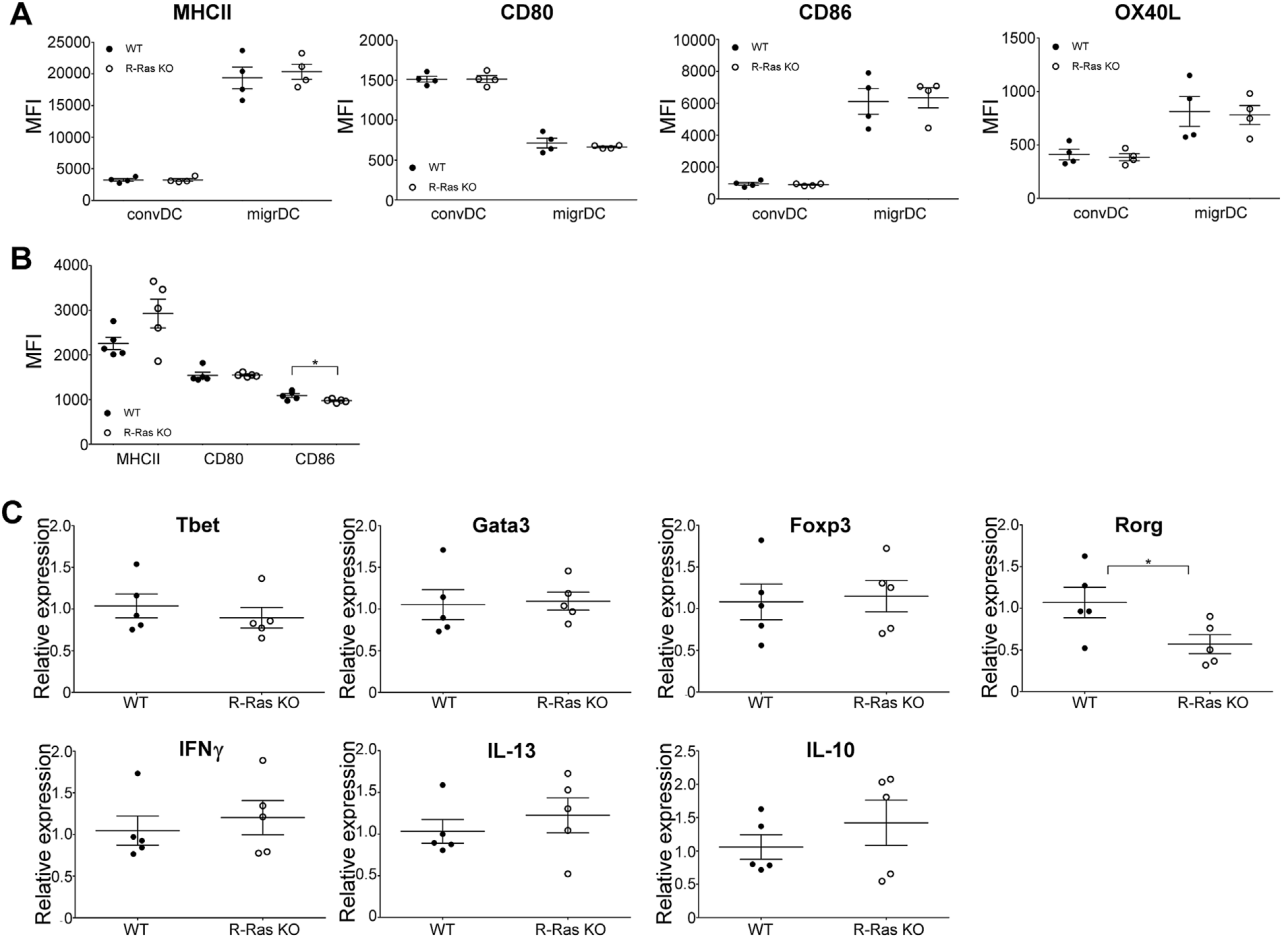
The expression of dendritic cell surface markers and mRNA expression of T‐helper cell transcription factors and cytokines after papain‐immunization in WT and R‐Ras KO mice. (A) LNs were collected from papain‐immunized (day 16) wild type (WT, *n* = 4) or R‐Ras deficient (R‐Ras KO, *n* = 4) mice and dendritic cells were enriched by magnetic separation. The expression of different surface proteins (MHCII, CD80, OX40L, and CD86) was investigated directly after euthanizing the mice. (B) Spleens were collected from papain‐immunized (day 16) wild type (WT, *n* = 5) or R‐Ras deficient (R‐Ras KO, *n* = 5) mice and dendritic cells were enriched by gradient centrifugation. The expression of different surface proteins (MHCII, CD80, and CD86) was analyzed with flow cytometer. The differences within surface protein expression were statistically analyzed (unpaired two‐tailed *t*‐tests. *p < 0.05). (C) Spleens were harvested from day 16 papain‐immunized wild type (WT, *n* = 5) and RAS‐deficient (R‐Ras KO, *n* = 5) mice and prepared as in Figure 2. qPCR analysis for T cell transcription factors and cytokines was performed. Each dot represents an individual animal. Mean values with SEM are marked in the scatter dot plots. The differences within gene expression values were statistically analyzed by two‐tailed unpaired *t*‐tests. *p < 0.05

Then we analyzed the expression of DC activation markers from papain‐immunized mice. Unlike in the healthy mice, the conventional and migratory DC's from the LN showed no difference between the two genotypes in the expression of MHCII, CD80, CD86, or OX40L (Fig. 4A). In splenic DC's the expression of MHCII and CD80 were similar between WT and R‐Ras KO animals, but CD86 expression was reduced after papain‐immunization in R‐Ras KO mice (Fig. 4B). Papain has been shown to induce Th2 polarization in multiple studies, determined by the IL‐4 production of T cells 17, 28, 30 and T cell recall responses upon in vitro restimulation with papain 17, 28, 31, 32. We measured the mRNA expression of transcription factors associated with T cell differentiation from splenocytes by qPCR after papain‐immunization. The mRNA expression levels of Tbet, GATA3, and Foxp3 were identical between WT and R‐Ras KO mice but for Rorγ, the master regulator of Th17‐differentiation, the mRNA expression was significantly lower (*p* = 0.0492) in the R‐Ras KO mice (Fig. 4C). Accordingly, hallmark cytokine mRNA analysis from these samples indicated no difference between IFN‐γ, IL‐13, or IL‐10 expression (Fig. 4C). The notion that RORγ‐transcription factor was expressed at reduced levels in R‐Ras KO mice compared to WT is logical as IL‐17 production is decreased in CNS of *Rras^−/^* ^−^ mice 15 undergoing EAE or carcinogen induced tumor development 13. RORγ, on the other hand, plays a critical role in the development of IL‐17 producing CD4+ T cells 33. Also, a recent study showed that epicutaneous papain administration was able to induce the differentiation of allergen‐specific Th17 cells 31, suggesting that papain induces not only Th2 but also Th17 responses.

To our knowledge, this is the first study that addresses the effect of R‐Ras on allergen‐induced immune response. Although we could not demonstrate any role for R‐Ras in regulating the immune response toward papain‐immunization, future work utilizing IL‐4‐dependent, and IL‐4‐independent Th2 polarizing models (such as *Trichurus muris* and *Nippostrongylus brasiliensis* infections) could specify a more defined role for R‐Ras in immune response.

## Authors’ Contributions

T.J., I.J., L.K., Z.O., S.P., M.V., and H.U‐J. designed the research. L.K., Z.O., and M.V. performed the experiments. L.K., Z.O., M.V., S.P., H.U‐J., T.J., and I.J analyzed the data. H.U.J. and M.V. contributed the genotyped mice littermates. L.K., Z.O., T.J., and I.J. wrote the manuscript.

## Conflict of Interest

The authors declare no conflict of interest.

## Supporting information

Additional supporting information may be found in the online version of this article at the publisher's web‐site.

**Figure S1**. Gating strategies used in flow cytometry. (A) Spleens were harvested from euthanized mice. Single cell suspension was created as described in Materials and Methods and red blood cells lysed before staining the cells for surface markers and applying them onto the flow cytometer. Cell debris was excluded and remaining cells were studied for CD3 and B220 expression, and T cells were gated as CD3^+^ population (T cell) and B cells as B220^+^ group (B cell). T cells were further analyzed for CD4 and CD8 surface marker expression, and CD4^+^CD8^−^ and CD8^+^CD4^−^ subpopulation were identified as CD4+ T cells (CD4+) and CD8+ T cells (CD8+), respectively. (B) Strategy for IL‐33R gating in CD4 cells. (C) For LN DC's, single cell suspensions of LN's were prepared as in Figure 1. CD11c^+^MHCII^+^ dendritic cells involve two subpopulations: conventional dendritic cells (convDC) show higher CD11c and lower MHCII expression while migratory dendritic cells express less CD11c but more MHCII. (D) For splenic DC's, spleens were prepared as described in Materials and Methods. Cell debris was excluded and cells were investigated for the expression of CD3 (T cell marker), B220 (B cell marker) and F480 (macrophage marker). CD3^−^B220^−^F480^−^ cells (lin.neg.) were then studied further for the expression of CD11c and MHCII. The figure shows the gating of one representative sample for each case.
**Figure S2**. Expression of DC surface markers in WT and R‐Ras KO splenic DC's in response to various stimulants. Spleens were prepared as described in materials and methods from WT (*N* = 3) an R‐Ras KO (*n* = 3) mice. The cells were either stained directly (ex vivo) or after o/n culture with medium alone or with poly(I:C), LPS, or IL‐4 as indicated. The expression of MHCII (A), CD80 (B), OX40L (C), and CD86 (D) was measured with flow cytometer.
