# Supplementary material for: R‐Ras deficiency does not affect papain‐induced IgE production in mice
Source: Immun Inflamm Dis. 2017 May 11;5(3):280–8. doi: 10.1002/iid3.168 (PMC5569372; doi:10.1002/iid3.168)
Supplement: Supplementary file 1 — Figure S1. Gating strategies used in flow cytometry. (A) Spleens were harvested from euthanized mice. Single cell suspension was created as described in Materials and Methods and red blood cells lysed before staining the cells for surface markers and applying them onto the flow cytometer. Cell debris was excluded and remaining cells were studied for CD3 and B220 expression, and T cells were gated as CD3+ population (T cell) and B cells as B220+ group (B cell). T cells were further analyzed for CD4 and CD8 surface marker expression, and CD4+CD8− and CD8+CD4− subpopulation were identified as CD4+ T cells (CD4+) and CD8+ T cells (CD8+), respectively. (B) Strategy for IL‐33R gating in CD4 cells. (C) For LN DC's, single cell suspensions of LN's were prepared as in Figure 1. CD11c+MHCII+ dendritic cells involve two subpopulations: conventional dendritic cells (convDC) show higher CD11c and lower MHCII expression while migratory dendritic cells express less CD11c but more MHCII. (D) For splenic DC's, spleens were prepared as described in Materials and Methods. Cell debris was excluded and cells were investigated for the expression of CD3 (T cell marker), B220 (B cell marker) and F480 (macrophage marker). CD3−B220−F480− cells (lin.neg.) were then studied further for the expression of CD11c and MHCII. The figure shows the gating of one representative sample for each case. Figure S2. Expression of DC surface markers in WT and R‐Ras KO splenic DC's in response to various stimulants. Spleens were prepared as described in materials and methods from WT (N = 3) an R‐Ras KO (n = 3) mice. The cells were either stained directly (ex vivo) or after o/n culture with medium alone or with poly(I:C), LPS, or IL‐4 as indicated. The expression of MHCII (A), CD80 (B), OX40L (C), and CD86 (D) was measured with flow cytometer. [file IID3-5-280-s001.pdf]

SUPPLEMENTAL INFORMATION FOR MANUSCRIPT: "R-RAS deficiency does not affect papain-induced IgE production in mice"

Laura Kummola, Zsuzsanna Ortutay, Maria Vähätupa, Stuart Prince, Hannele Uusitalo-Järvinen, Tero AH Järvinen, Ilkka S Junttila

SUPPLEMENTARY FIGURES

Supplementary figure 1. Gating strategies used in flow cytometry. (A) Splenic single cell suspension was created as described in Methods. Cell debris was excluded and remaining cells were studied for CD3 and B220 expression. T cells were gated as $CD3^+$ population (T cell) and B cells as $B220^+$ group (B cell). T cells were further analyzed for CD4 and CD8 expression, and $CD4^+CD8^-$ and $CD8^+CD4^-$ subpopulation were identified as CD4+ T cells (CD4+) and CD8+ T cells (CD8+). (B) Strategy for IL-33R gating in CD4 cells. (C) LN's were prepared as in Fig1. $CD11c^+MHCII^+$ dendritic cells involve two subpopulations: conventional dendritic cells (convDC) show higher CD11c and lower MHCII expression while migratory dendritic cells express less CD11c but more MHCII. (D) For splenic DC's were prepared as described in Methods. Cell debris was excluded and cells were investigated for the expression of CD3 (T cell marker), B220 (B cell marker) and F480 (macrophage marker). $CD3^-B220^-F480^-$ cells (lin.neg.) were then studied further for the expression of CD11c and MHCII. The figure shows the gating of one representative sample for each case.

Supplementary Figure 2. Expression of DC surface markers in WT and R-Ras KO splenic DC's in response to various stimulants. Spleens were prepared as described in materials and methods from WT (N=3) an R-Ras KO (n=3) mice. The cells were either stained directly (ex vivo) or after o/n

culture with medium alone or with poly(I:C), LPS or IL-4 as indicated. The expression of MHCII (A), CD80 (B), OX40L (C) and CD86 (D) was measured with flow cytometer.

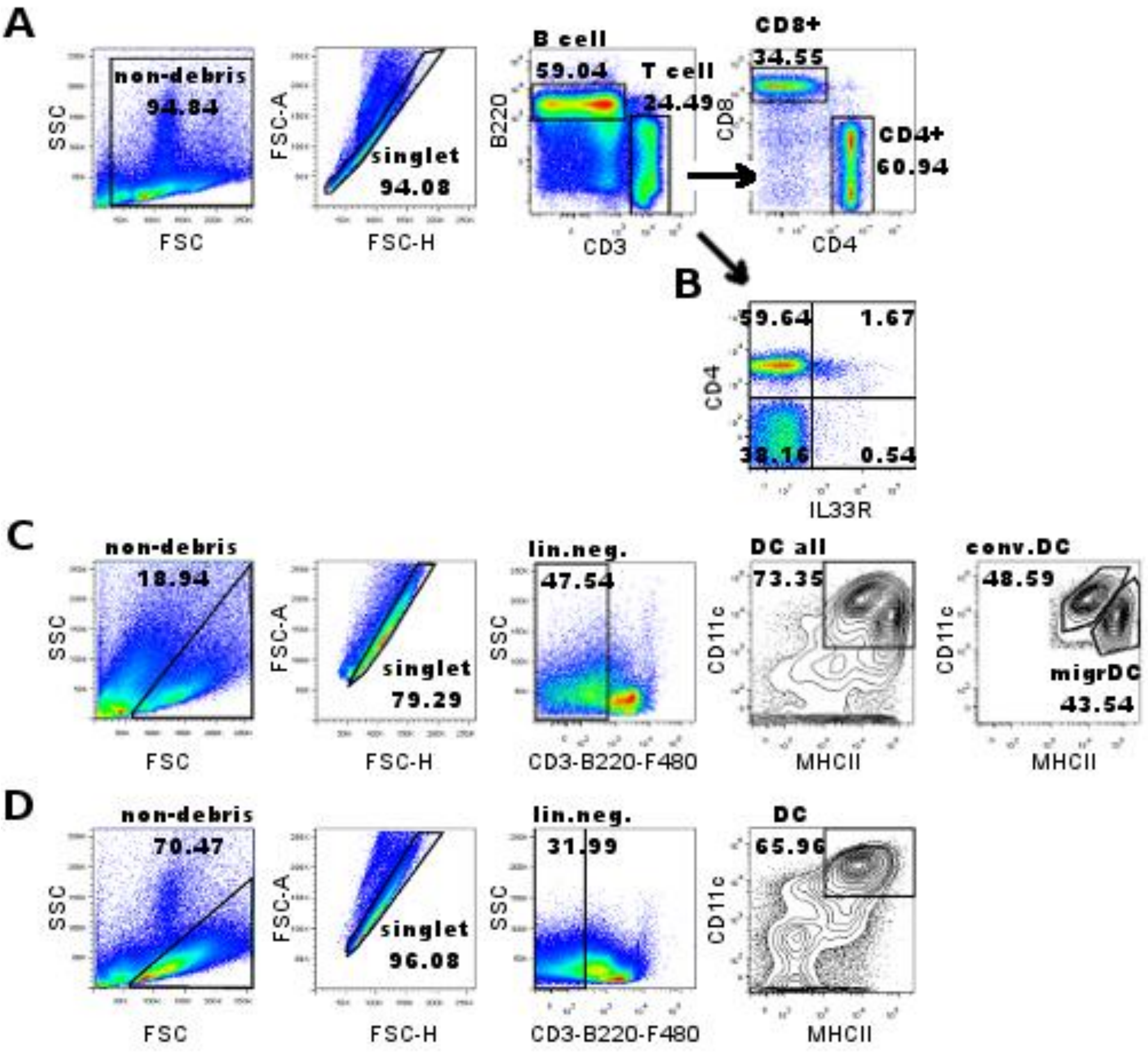

Supplementary figure 1: Gating strategy

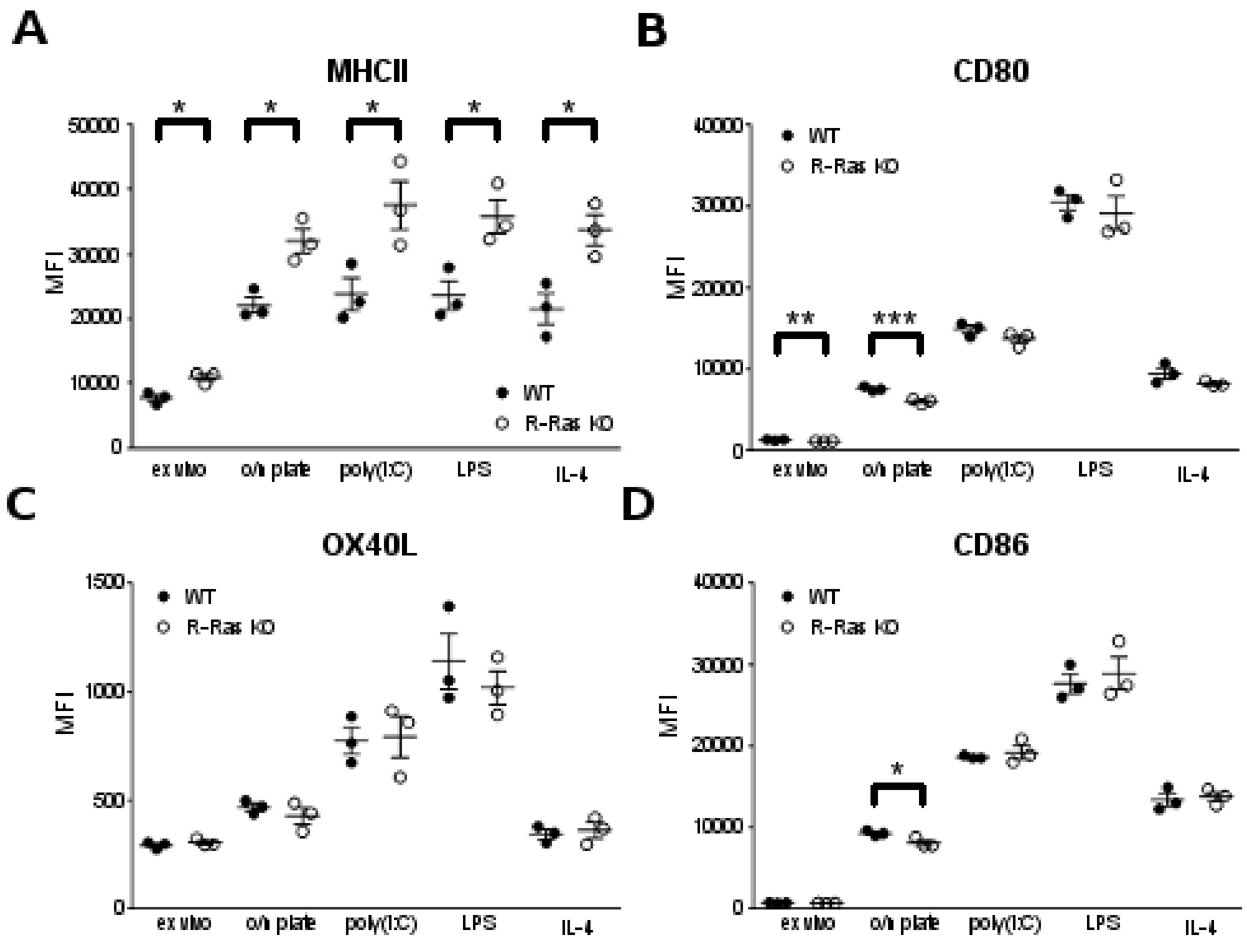

Supplementary figure2: Changes in surface markers of splenic dendritic cells after in vitro stimulation
